# Research agenda for ending preventable maternal deaths from postpartum haemorrhage: a WHO research prioritisation exercise

**DOI:** 10.1136/bmjgh-2024-015342

**Published:** 2024-11-07

**Authors:** Caitlin R Williams, Guervan Adnet, Ioannis D Gallos, Arri Coomarasamy, A Metin Gülmezoglu, Md Asiful Islam, Sara Rushwan, Mariana Widmer, Fernando Althabe, Olufemi T Oladapo

**Affiliations:** 1 Department of Mother and Child Health, Institute for Clinical Effectiveness and Health Policy, Buenos Aires, Argentina; 2 Boston Consulting Group, Paris, France; 3 UNDP/UNFPA/UNICEF/WHO/World Bank Special Programme of Research, Development and Research Training in Human Reproduction (HRP), Department of Sexual and Reproductive Health and Research, World Health Organization, Geneva, Switzerland; 4 WHO Collaborating Centre for Global Women’s Health, Institute of Metabolism and Systems Research, College of Medical and Dental Sciences, University of Birmingham, Birmingham, UK; 5 Concept Foundation, Geneva, Switzerland

**Keywords:** Obstetrics, Maternal health, Decision Making, Other study design

## Abstract

**Introduction:**

Postpartum haemorrhage (PPH) remains the leading cause of maternal death. Yet there is a lack of clarity around what research is needed to determine what works and how best to deliver proven PPH interventions. This article describes a WHO-led effort to develop a global PPH research agenda for 2023–2030, to reinvigorate research and innovation while avoiding duplication and waste.

**Methods:**

Potential questions were culled from evidence gaps in a forthcoming Lancet PPH series, a pipeline analysis on PPH medicines and devices, international PPH guidelines, previous research prioritisation efforts and submissions from a reference group of PPH experts and stakeholders. Questions were deduplicated and consolidated, categorised into three tracks (innovation, implementation and cross-cutting) and subjected to an online prioritisation survey. Survey participants (n=120) assessed these questions using five criteria (answerability, effectiveness, deliverability, maximum potential for disease burden reduction and equity) following the Child Health and Nutrition Research Initiative methodology. The outcome of this exercise was complemented by an in-person consensus meeting (Global PPH Summit from 7 March 2023 to 10 March 2023 in Dubai, United Arab Emirates) to finalise the research agenda.

**Results:**

Fifteen research questions (five per track) were identified as top priority. The top question per track called for research on the comparative effectiveness and safety of alternative routes of administration (other than the intravenous route) of tranexamic acid in the treatment of PPH (innovation); identifying barriers and facilitators affecting the adoption and use of evidence-based recommendations for PPH management (implementation) and the effectiveness of a strategy of early detection and first response treatment using a bundle of recommended interventions for improving PPH-related outcomes (cross-cutting).

**Conclusion:**

This shared research agenda should guide future investments into PPH studies with high potential to transform policy and clinical practice in the near term to medium term. Funding for the new research priorities is urgently needed.

**WHAT IS ALREADY KNOWN ON THIS TOPIC**

- In a 2015 maternal health research prioritisation exercise, WHO identified three priority questions specific to PPH among a total of 20 priority questions. The broader maternal health scope may have obscured potentially important questions for advancing efforts to address PPH, and many PPH-specific questions remain unanswered. To date, no global PPH-specific research prioritisation exercise has been conducted.

**WHAT THIS STUDY ADDS**

- This article describes a WHO-led process to develop a global research agenda for PPH and presents the 15 top priority research questions.

**HOW THIS STUDY MIGHT AFFECT RESEARCH, PRACTICE OR POLICY**

- By delineating research priorities, this exercise provides guidance for potentially high-impact research and development investments. It can help spur the development of implementation strategies to accelerate uptake of life-saving interventions. High-quality research responding to these priority questions can inform global and national policies that would support implementation and sustainability of programmes aimed at reducing mortality and morbidity from PPH.

## Introduction

 Postpartum haemorrhage (PPH) remains the leading direct cause of maternal death worldwide, accounting for an estimated 27% of the global burden of maternal mortality.^1 2^ Even women who survive PPH can face long-term morbidities, both physical and psychological. Long-term sequelae of PPH include anaemia, bladder injury, postpartum depression, sexual dysfunction and post-traumatic stress disorder.^3^,^7^ The women and communities most affected by PPH are already some of the most marginalised, underscoring the equity and human rights imperatives of ending preventable morbidity and mortality due to PPH.^8^

Given the substantial proportion of maternal deaths attributable to PPH, the global maternal health community needs to make significant progress in preventing PPH-related deaths to meet the Sustainable Development Goal 3.1 target. Critically, reducing deaths due to PPH would have a large impact in countries with some of the heaviest burdens of maternal death.^9^ A targeted focus on PPH could help to address issues of equity, by ensuring that the primary driver of morbidity and mortality in the most affected communities is at the top of the political agenda.

Despite a clear case for ending preventable deaths due to PPH, progress has been slow. Trend data show that reductions in maternal deaths have plateaued in recent years.^8^ Although research is crucial for developing innovative solutions and finding better ways to implement effective solutions, the past decade has witnessed much research duplication and waste.^10^ Ensuring the global maternal health community has a clear research agenda and central mechanism for organising and coordinating efforts around that agenda can help to streamline efforts and propel the field forward.

In March 2023, the UNDP/UNFPA/UNICEF/WHO/World Bank Special Programme of Research, Development and Research Training in Human Reproduction, Department of Sexual and Reproductive Health and Research, WHO, convened the first Global Summit on Postpartum Haemorrhage (hereafter, PPH Summit) to develop a common agenda and galvanise action on PPH. At the PPH Summit—held from 7 March 2023 to 10 March 2023 in Dubai, United Arab Emirates—participants gathered to develop a common agenda around four strategic areas: research, norms and standards, implementation and advocacy. Together, these four strategic areas contribute to a single unified Roadmap and Call to Action on PPH for the global maternal health community.^11^

The objective of the research strategic area was to align around priority research gaps that will accelerate progress and lead to the development of innovations that can engender sustained reduction in the burden of PPH and related adverse outcomes. This article describes the process and resulting common research agenda for PPH for the coming years.

## Methods

Prior to the start of the research prioritisation effort, an 18-member steering committee was established by WHO to advise the WHO Secretariat on the PPH Summit. The steering committee members were global thought leaders who were selected at the sole discretion of WHO, taking into account the following (non-exclusive) criteria: relevant scientific and technical expertise in PPH prevention and treatment strategies; experience in international and country policy development and advocacy work; direct, practical and in-depth country experience from research to impact assessment; excellent communication skills and fluency in English language; evidence of strong planning, organisational and analytical skills; demonstrated ability to manage converging priorities and build consensus; and ability to work constructively with people from different cultural backgrounds and orientations. The names of potential steering committee members were obtained through a request for nomination to WHO research and implementation partners, industry and private sector experts, donor community, non-governmental organisations, professional associations and UN Agencies, working in the field of PPH. Self-nominations were not permitted. The selection considered the composition of the steering committee as a whole, taking into account and prioritising the need for diverse perspectives through a balance of geographical representation, gender, technical expertise and diversity of experience and professional background, and with representation across predefined stakeholder groups—academic researchers, guideline development and content experts, industry experts and private sector working (or anticipated to be working) in the PPH space, international professional associations and their key affiliated societies, non-governmental organisations and civil society, international donor agencies, country ministries of health, and UN Agencies and their partnerships. Members served in their personal capacity. Steering committee members then nominated individuals across the above-mentioned stakeholder groups to participate in the agenda setting for the four strategic areas at the PPH Summit. The final list of PPH Summit participants was reviewed and agreed by the WHO Secretariat and Steering Committee, adhering to the same principles to ensure diversity through geographic, gender and technical expertise balance in accordance with WHO policy. For the research agenda-setting effort, the steering committee provided guidance on the methods for identifying and prioritising key research gaps, ensuring that the methods were rigorous and compliant with WHO internal procedures, and along with the PPH Summit participants executed the research prioritisation exercise.

**Figure 1:**
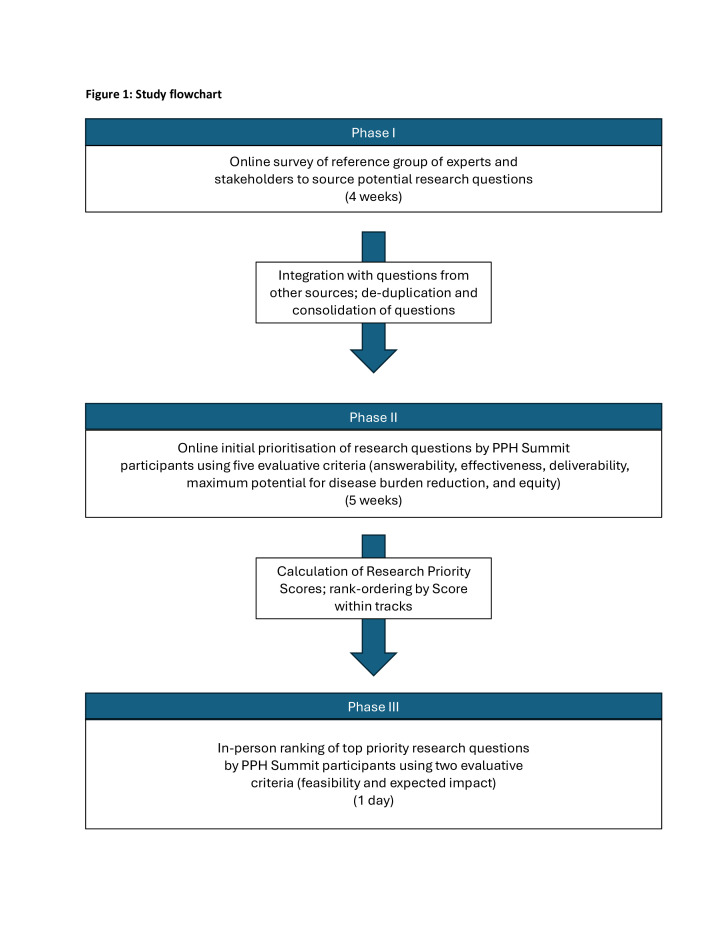
Study flow chart. PPH, postpartum haemorrhage.

Following guidance from the steering committee, the research prioritisation effort was designed using the Child Health and Nutrition Research Initiative (CHNRI) methodology,^12^,^14^ with an additional final in-person stakeholder meeting to build consensus around the final set of research priorities. The CHNRI methodology was selected over the Delphi method due to the efficiency of generating a rank-ordered list of priorities (ie, it requires a single round of ranking rather than multiple iterative surveys that can contribute to respondent fatigue and low response rates), as well as its flexibility in engaging non-experts in the priority-setting process. In addition, the CHNRI methodology does not require participants to review a set of prereads in advance of participating.^13 14^ The goal was to identify and prioritise research knowledge gaps to address PPH burden and complications in the short, medium and longer terms. This entire process was managed by WHO and implemented in three phases: (1) generation, collection and consolidation of research questions; (2) prioritisation of research questions using a scoring system based on five criteria and (3) determination of the final list of research priorities. The overall structure of the prioritisation exercise is depicted in figure 1.

### Phase I

The first phase focused on developing a comprehensive list of current evidence gaps related to PPH, where additional research could help to move the field forward. These gaps were formulated as broad possible research questions, which could then be prioritised. Research questions were identified from multiple sources: evidence gaps from a comprehensive evidence synthesis conducted by an international group of experts commissioned by the Lancet to produce a series on PPH (under development), a pipeline analysis of medicines and devices related to PPH,^15^ research priorities included in guidelines published by WHO and other international guideline developers,^16^,^31^ unaddressed questions from the 2015 WHO maternal health research prioritisation exercise,^32^ and an open call for research questions sent to a reference group of experts and stakeholders.

From the list of potential PPH Summit participants already identified, the steering committee and WHO Secretariat selected a reference group of 106 PPH experts and stakeholders. Gender and geographical balance were ensured. The WHO Secretariat then invited this reference group to provide input to the list of research questions to be considered for prioritisation. All members of the reference group were invited to electronically provide a maximum of three research questions related to PPH over a period of 1 month. 74 of the reference group members responded positively to the invitation.

Research questions from all sources were consolidated into a single list. The WHO Secretariat independently reviewed all questions on the list to identify and exclude duplicate questions, questions that were out of scope (ie, not pertaining to PPH), and questions that were too broad (eg, ‘research to reduce PPH-related maternal mortality’, ‘develop and test interventions for reducing PPH’). A reduced list of questions was then subjected to thematic analysis. The thematic analysis consisted of grouping similar questions together to identify research themes and subthemes, which allowed for the identification of additional duplicates and out-of-scope questions. Questions were edited for clarity and similar questions were merged. During this process, questions were edited to achieve a level of detail compatible with the CHNRI concept of ‘research avenues’ (ie, a research question that is neither too broad nor too specific and can be answered through a set of individual research projects); hence, very detailed and specific questions were made more general.^13^ Once the questions were formulated, they were categorised into one of three tracks (innovation, implementation and cross-cutting), which map onto the CHNRI 4D’s framework (table 1).

**Table 1:** Mapping of CHNRI constructs into PPH research prioritisation tracks

Research domain	Research avenues	CHNRI ‘4D’ framework	PPH Summit tracks
Research to assess burden of PPH and its determinants	Measuring the burden of PPH, morbidity, mortality Understanding risk factors associated with PPH Measuring prevalence of exposure to risk factors for PPH Evaluating the efficacy and effectiveness of available interventions Measuring prevalence of coverage of interventions in place	Description Development	Cross-cutting
Research to develop new capacities to reduce the burden of PPH	Basic, clinical and public health research to explore entirely novel ideas to develop new capacities for managing PPH Basic, clinical and public health research to advance on existing knowledge to develop new capacities for managing PPH	Discovery	Innovation
Health research to improve performance of existing capacities to reduce the burden of PPH	Improving existing PPH detection, prevention and treatment interventions (their affordability, deliverability) Health policy analysis relating to PPH Health system structure analysis relating to PPH Financing/costs analysis relating to PPH Human resources relating to PPH Provision/infrastructure relating to PPH Implementation research relating to PPH Responsiveness/recipients acceptability of interventions/strategies relating to PPH	Delivery	Implementation

CHNRI, Child Health and Nutrition Research Initiative; PPH, postpartum haemorrhage.

### Phase II

In phase II, the reduced list of questions (online supplemental file S1) was shared with 126 participants who had been selected to attend the PPH Summit (some of whom participated as members of the reference group in phase I) for an initial round of prioritisation. The distribution of these participants across the predefined stakeholder groups is as follows: academic researchers (n=18), guideline development and content experts (n=20), industry and private sector experts (n=13), professional associations (n=13), non-governmental organisations and civil society (n=16), country ministries of health (n=18), international donor agencies (n=13), and UN Agencies and their partnerships (n=15). The above categorisation into stakeholder groups was based on the attribute that best described participant’s area of work, as the scope of work of several participants cut across multiple stakeholder groups. The number of participants in each stakeholder group was fairly balanced to ensure that no stakeholder group dominated the agenda-setting activity. In advance of the PPH Summit, these 126 participants were sent a unique link to an online survey (via SurveyMonkey) in which they would assess each of the proposed research questions. Completion and submission of the survey indicated consent to participate. A total of 120 invited participants completed the survey. Six did not respond to the survey and did not offer any reasons. There were no discernible patterns among the survey non-respondents. All respondents participated in their own individual capacity and not as representatives of their organisations.

The survey was divided into innovation, implementation and cross-cutting tracks. Participants could choose the order in which they wanted to address the tracks. They also had the option to skip tracks if they felt they were not knowledgeable enough to provide a reasonable assessment. Participants were asked to assess each question with respect to five criteria: answerability, effectiveness, deliverability, maximum potential for disease burden reduction and equity (table 2). These criteria are described in detail in the CHNRI guidelines.^12^ The survey was open for 5 weeks (25 January 2023–1 March 2023).

**Table 2:** Scoring criteria for setting research priorities

Criteria	Definition
Answerability	The research question can be ethically answered.
Effectiveness	The new knowledge is likely to result in an effective intervention or programme.
Deliverability	The intervention or programme will be deliverable, acceptable and affordable.
Potential impact	The intervention or programme has the potential to substantially reduce maternal and perinatal mortality, morbidity and long-term disabilities.
Equity	The intervention or programme will reach the most vulnerable groups.

For each criterion, participants were asked to answer either yes, no, don’t know or no opinion. Following CHNRI procedures, responses were assigned a corresponding numeric score (yes=1, no=0, don’t know=0.5), with ‘no opinion’ responses being removed from the analysis entirely (ie, respondent was dropped from the denominator). All criteria were weighted equally. Reponses were aggregated into interim scores by criterion and then final Research Priority Scores (RPS). Interim scores by criterion were calculated as simple arithmetic means (sum of points assigned during scoring/total number of responses). As all criteria were weighted equally, the final RPS was calculated as a simple arithmetic mean of the interim scores. A mathematical formula for the generalised CHNRI scoring is:

RPS = W_1_(P_1_/T_1_)+W_2_(P_2_/T_2_)+…+W_n_(P_n_/T_n_)

The list of questions was ranked by RPS both overall and within each track.

### Phase III

The methods and top 10-ranked questions in each track were shared at the PPH Summit during a plenary session. After the presentation, participants were split evenly into three breakout groups corresponding to the three tracks, to reach consensus on the top five highest priority research questions. Composition of the breakout groups was intentional to ensure a balance between gender, geographical region and type of participant (ie, ministries of health, UN agency, donor, etc). Technical briefs were presented to provide additional context and background information. One technical brief was prepared for each of the 30 questions so breakout sessions were presented with 10 technical briefs each (online supplemental file S2). After reviewing the technical briefs, breakout groups discussed the feasibility and expected impact of the 10 prioritised questions for their track. To keep discussions focused, given the large number of breakout group participants, it was determined that only one or two criteria should be considered during discussion. Feasibility of conducting research that could answer the research question was deemed to be an important additional criterion to consider, with expected impact as a critical (and potentially contradictory) criterion. Following the open discussion, each participant independently ranked their top five research questions through the online Slido voting platform in real time.^33^ Results were aggregated to provide a group ranking of the top five highest priority questions, which would go on to be designated as part of the research agenda for the period 2023–2030 (and beyond for innovation). Importantly, research questions with studies currently underway but not yet completed were still considered, as those research questions were still unanswered at the time of the prioritisation.

### Patient and public involvement

Given the technical nature of the exercise, it was deemed inappropriate to involve patients or the public in the design, conduct and reporting of this prioritisation exercise. However, it was recognised that some participants were mothers who had given birth (including some who had personally experienced PPH) in addition to being expert stakeholders in PPH in a professional capacity. Representatives of women’s organisations constituted one of the key expert stakeholder groups involved in the prioritisation exercise, to ensure women’s experiences and perspectives on PPH were included in the process.

## Results

### Phase I

**Figure 2:**
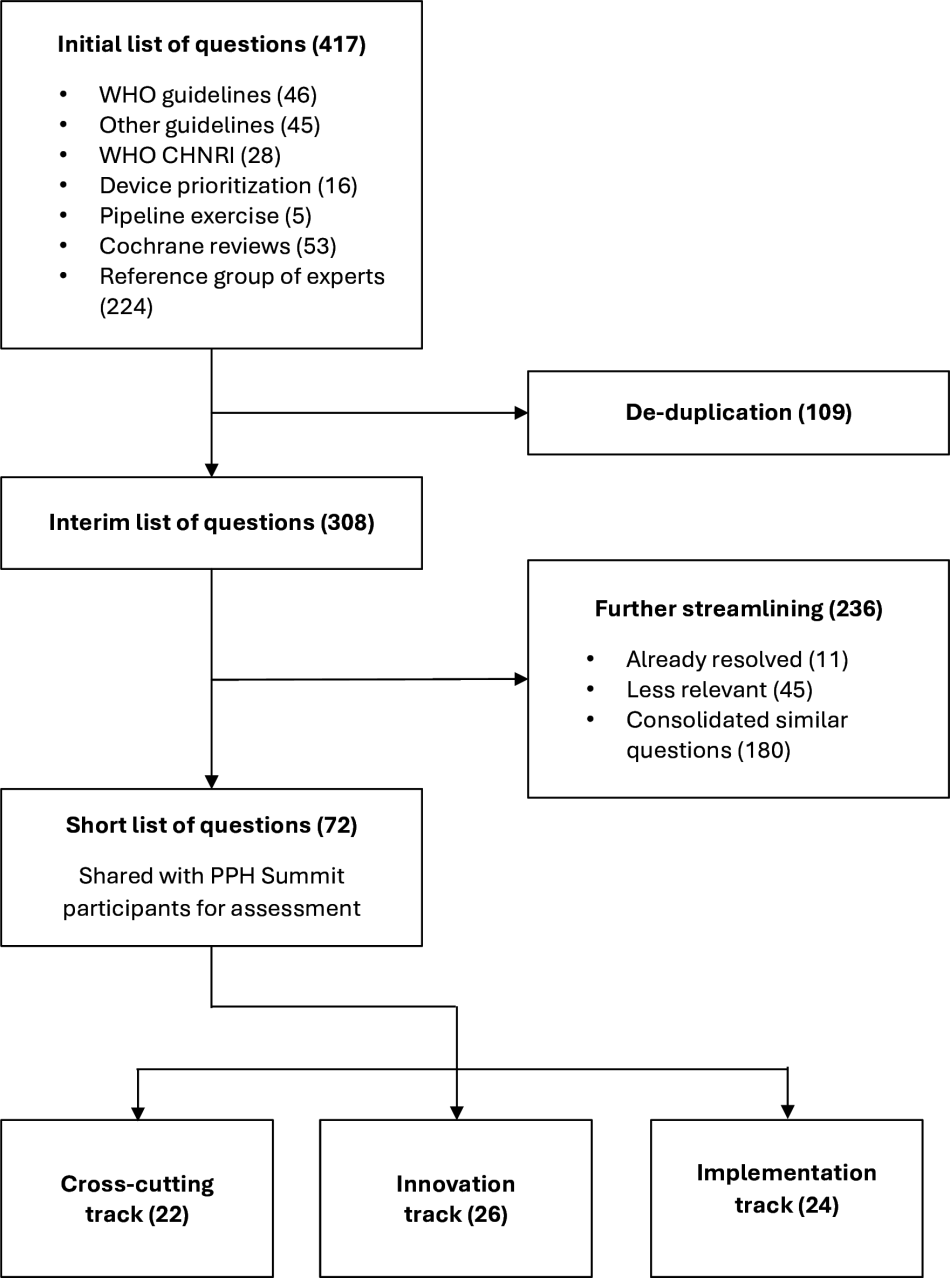
Flow chart for developing the short list for scoring. CHNRI, Child Health and Nutrition Research Initiative; PPH, postpartum haemorrhage.

From the initial sourcing of potential research questions, 417 questions were identified. Following review, consolidation and editing, this was reduced to a final list of 72 research questions (figure 2). Of those questions, 22 were in the cross-cutting track, 26 were in the innovation track and 24 were in the implementation track.

### Phase II

The overall response rate for the survey was 95.2% (120 respondents out of 126 invited to participate). No specific patterns were observed among non-respondents with respect to profession, gender or geography. The full list of questions ranked in order of RPS scores is provided in online supplemental file S1.

### Phase III

The final top five priorities from each breakout group are provided in table 3.

**Table 3:** Final top priority research questions by track, ordered from highest to lowest priority

Rank	Innovation	Implementation	Cross-cutting
1	What is the comparative effectiveness and safety of alternative routes of tranexamic acid (TXA) in the treatment of PPH?	What are the implementation barriers and facilitators affecting the adoption and use of evidence-based recommendations for PPH management?	What is the effectiveness of a strategy of early detection and first response treatment using a bundle of recommended interventions for improving PPH-related outcomes?
2	What is the effectiveness and safety of heat-stable carbetocin for PPH treatment in women who received heat-stable carbetocin for PPH prevention?	What are the optimal strategies to ensure access to quality-assured PPH medicines (including universal health coverage/essential packages for health services and health benefit package) in low-income and middle-income countries?	What is the effectiveness and safety of a diagnostic algorithm (eg, Shock Index) and early detection strategies (eg, Modified Early Obstetric Warning Score) in improving clinical detection and management of PPH?
3	What is the comparative effectiveness of uterine balloon tamponade devices compared with other tamponade interventions (such as suction devices) in the reduction of PPH-related maternal morbidity and mortality?	What are the most effective advocacy strategies to improve the uptake and ensure sustainment of evidence-based practices for PPH management at the country level?	What is the effectiveness of checklists in improving PPH quality of care and PPH-related outcomes compared with current standard of care?
4	Can clinical criteria for haemodynamic instability facilitate earlier PPH diagnosis and improved PPH outcomes compared with blood loss measurement alone?	What is the effectiveness and cost of pre-service and in-service training programmes for front-line healthcare workers (paramedics, general practice doctors, community health workers, midwives, nurses) to manage and refer women with PPH?	What is the effectiveness of Maternal and Perinatal Death Surveillance and Response programmes in the reduction of maternal deaths due to PPH?
5	What strategies are most effective for engaging the private sector in the development of new PPH medicines, devices and diagnostics in low-income and middle-income countries?	What are the most effective implementation strategies to improve uptake and sustainment of recommended evidence-based interventions for PPH management, including in humanitarian settings?	What is the effectiveness and safety of TXA in the prevention of PPH in general obstetric population and in women at high risk of PPH (eg, anaemic women)?

PPH, postpartum haemorrhage.

## Discussion

This article outlined the process that led to the identification of 15 top priority PPH research areas across three tracks (innovation, implementation and cross-cutting), corresponding to three research domains. Each of the three research domains consisted of multiple research avenues, as depicted in table 1. However, the final top 15 prioritised research questions clustered around just a few research avenues: evaluating the efficacy and effectiveness of available interventions; basic, clinical and public health research to advance on existing knowledge to develop new capacities for managing PPH; and implementation research regarding PPH. Some of this clustering already emerged from the list of questions received from members of the reference group in phase I. For example, there were few questions about the burden of PPH or the risk factors associated with PPH (see full list of questions in online supplemental file S1). However, the clustering may have been intensified by the choice of evaluative criteria used in phases II and III. The factors ‘feasibility’ and ‘potential for impact’ stressed in phase III may have influenced participants toward prioritising research questions that would apply existing interventions to improve implementation and uptake rather than novel interventions. Proposed implementation research questions were largely focused on the implementation outcome of uptake/adoption, rather than acceptability, cost or sustainability.^34^ Implementation research centred on some of these other implementation outcomes could also have helped to address other research avenues within the ‘Health research to improve performance of existing capacities to reduce the burden on PPH’ research domain. In addition to the top-ranked questions that form the basis of the common research agenda, this process led to the identification and prioritisation of numerous other research questions, which can feed subsequent waves of research focus as questions in the top 15 are addressed.

This work builds on a maternal and perinatal health previous research prioritisation,^32^ but intentionally sought to canvas a large and diverse set of stakeholders to align around a common research agenda specific to PPH. The earlier research prioritisation exercise, conducted in 2013 for the period 2015 to 2025, established broad research priorities across maternal and perinatal health. That exercise led to the prioritisation of three PPH-specific questions: (1) evaluate the effectiveness and cost of training interventions for frontline healthcare workers (paramedics, doctors, community health workers (CHWs), midwives, nurses) to diagnose, manage and refer women with obstetric haemorrhage; (2) develop and evaluate the effectiveness and cost of strategies to improve access of women with obstetric haemorrhage to blood and blood replacement products in settings without transport capabilities and (3) develop and evaluate the effectiveness of strategies to increase access of women to misoprostol at community level where oxytocin is not available/feasible, by dispensing it antenatally as part of a birthing kit, or at the time of delivery via the attending CHW or nurse/midwife, to prevent and treat PPH.^32^ The first question has not yet been answered and was revalidated as a key research priority through our PPH-focused research prioritisation exercise. The second question also remains unaddressed (a more detailed version of this question was ranked #28 in terms of priority in our exercise; the lower priority ranking may be related to the perceived feasibility and impact of solutions to address this question, as well as evolution in the research community’s thinking about the relative priority of this question over the last decade). In contrast, the third question had been addressed,^35^ and the research output underpins the subsequent WHO recommendation on advance distribution of misoprostol to pregnant women for prevention of PPH.^36^ The persistence of research questions across the two prioritisation exercises both highlights the importance of answering these questions and underscores the need for focusing on the maternal health research agenda to drive progress on the central questions facing the field. However, the PPH-focused research prioritisation exercise also surfaced numerous additional research priorities, which might have been obscured by a broader approach.

Of note, a comparison of the initial unprioritised list of PPH-related questions posed to participants in 2013 exercise with the list generated through our process in 2023 surfaces evidence of progress, evolving thinking and missed opportunities. Open research questions from the 2013 exercise about alternatives to oxytocin (carbetocin, ergometrine, misoprostol, a yet-to-be-developed heat-stable uterotonic), tranexamic acid for PPH treatment, uterine tamponade devices and standardised approaches to measuring blood loss to detect PPH all generated impactful evidence that was subsequently integrated into WHO recommendations.^30 31 37 38^ The variety of implementation-focused questions that arose in the new prioritisation exercise (as compared with a single question in 2013: ‘Evaluate the effectiveness and cost of guideline implementation strategies for managing obstetric haemorrhage at all levels of care to improve maternal and perinatal outcomes’) demonstrates the influence of advances within implementation science and implementation research. These newer questions are more focused and reflect an attention to implementation strategies and approaches, as well as the need to affect change at various leverage points across the health system. In addition, the growing interest in implementation-focused questions may be reflective of increasing sentiment that effective interventions exist but are underdeployed in many settings.^39 40^ Persistent questions regarding improving access to blood and blood products, developing diagnostic algorithms to improve PPH detection and determining which modalities of training offer the highest return spotlight the need for dedicated investments in these areas.

One of the strengths of this process was the intentionally broad and comprehensive approach to identifying possible research questions. In addition, the use of the CHNRI methodology ensured that the process used to rank and prioritise the research questions was rigorous, systematic and transparent. The prioritisation exercise involved a broad group of stakeholders (including those who invest in PPH research and those who promote and implement results of PPH research at the country level) to overcome the shortcomings of previously proposed methodologies driven by technical experts, where resultant research recommendations were rarely or not fully implemented.^12 32^ All stakeholders were given equal weight in voting, to prevent any group of stakeholders from having undue influence over the final research agenda. However, there were also limitations to this process. For example, though we sourced research questions from women’s representatives (eg, White Ribbon Alliance and WACI Health), the process could have benefited from more women’s groups to fully reflect the priorities of women and communities. Despite the rigorous facilitation of the breakout sessions, certain perspectives may have dominated group discussions in phase III. However, the anonymous and confidential nature of voting during this round of prioritisation helped mitigate pressure on participants to respond in a certain way during group discussions. Nonetheless, we acknowledge that this may not be sufficient to eliminate the potential for eminence or other messenger effects to sway participants’ responses. Still, this process and the results of the research prioritisation offer a common way forward for the global maternal health community in developing studies that will meaningfully advance the field.

## Conclusion

As outlined in the *Roadmap to Combat Postpartum Haemorrhage between 2023 and 2030*,^11^ these research priorities are intended to catalyse new investment in impactful research with high potential to change policy and practice. It is expected that funders will use this research agenda to develop and refine future commissioned calls, and that researchers will use the agenda to design impactful studies for grant applications, thus ensuring that research efforts are responsive to the most pressing questions in the field. To support these efforts, WHO will develop detailed Target Policy Profiles,^41^ which identify evidence gaps that must be addressed and the corresponding requirements for WHO to consider changes to its existing recommendations or the development of new ones. Target Policy Profiles will help funders commission calls for proposals that focus on the most meaningful pending questions, rather than funding studies that are inconsequential to the body of evidence underpinning future recommendations. Researchers will also benefit, by having a clearer understanding of the research priorities that have higher potential of influencing changes to policy and practice. WHO will continue to actively monitor the PPH research space and continuously respond to new, impactful evidence through its ‘living guidelines’ approach.^42^ Impactful evidence identified through an ongoing surveillance (‘intelligence gathering’) process will trigger the development or updating of recommendations and rapidly translate to policy and practice changes at the country level.

The establishment of a shared PPH research agenda presents a critical opportunity to steer future investments towards studies poised to make significant impacts on policy and clinical practice within the near term to medium term. This collective agenda will ensure that funding is directed towards research projects with the highest potential for driving meaningful improvement in maternal survival and well-being. The urgency for funding cannot be overstated in the light of the recently reported stagnation in the reduction of global maternal mortality ratio. Immediate funding support is required to initiate research initiatives to address identified priorities, as delays in investment could prolong the implementation of crucial findings and hinder progress towards reducing the burden of PPH-related mortality and morbidity worldwide.

## Supplementary material

10.1136/bmjgh-2024-015342 online supplemental file 1

10.1136/bmjgh-2024-015342 online supplemental file 2

## Data Availability

Data are available on reasonable request.
