# Supplementary material for: Research agenda for ending preventable maternal deaths from postpartum haemorrhage: a WHO research prioritisation exercise
Source: BMJ Glob Health. 2024 Nov 7;9(11):e015342. doi: 10.1136/bmjgh-2024-015342 (PMC11552529; doi:10.1136/bmjgh-2024-015342)
Supplement: online supplemental file 1 [file bmjgh-9-11-s001.pdf]

Supplementary File S1: Full list of questions assessed by PPH Summit participants, ranked by Research Priority Score (RPS)

Ranking	Track	Suggested research question	RPS
1	Implementation	What are the implementation barriers and facilitators affecting the adoption and use of evidence-based recommendations for PPH management?	99%
2	Implementation	What are the most effective advocacy strategies to improve the uptake and ensure sustainment of evidence-based practices for PPH management at the country level?	96%
3	Cross-cutting	What is the effectiveness of a strategy of early detection and first response treatment using a bundle of recommended interventions for improving PPH-related outcomes?	96%
4	Implementation	What are the most effective implementation strategies to improve uptake and sustainment of recommended evidence-based interventions for PPH management, including in humanitarian settings?	96%
5	Implementation	What are the optimal strategies to introduce and scale up the use of newer PPH medicines (e.g. heat-stable carbetocin, tranexamic acid) at various levels of care and settings?	96%
6	Innovation	What is the effectiveness and safety of heat-stable carbetocin for PPH treatment in women who received heat-stable carbetocin for PPH prevention?	95%
7	Implementation	What are the optimal strategies to ensure access to quality-assured PPH medicines (including Universal Health Coverage/Essential Packages for Health Services and Health Benefit Package) in low- and middle-income countries?	95%
8	Implementation	What are the critical components/models for successful introduction and implementation of new/newly recommended medications for PPH management in low- and middle-income countries?	95%
9	Implementation	What is the impact of training programmes for community health workers and paramedics on temporising measures (e.g. uterine massage, external aortic compression, uterine tamponade, Non-pneumatic Anti-Shock Garment) on PPH outcomes?	95%
10	Implementation	What is the effectiveness and cost of pre-service and in-service training programmes for frontline healthcare workers (paramedics, general practice doctors, community health workers, midwives, nurses) to manage and refer women with PPH?	94%
11	Cross-cutting	What is the effectiveness and safety of a diagnostic algorithm (e.g. shock index) and early detection strategies (e.g. Modified Early Obstetric Warning Score) in improving clinical detection and management of PPH?	93%
12	Innovation	What is the comparative effectiveness and safety of alternative routes of tranexamic acid (TXA) in the treatment of PPH?	93%

13	Cross-cutting	What is the effectiveness and safety of tranexamic acid (TXA) in the prevention of PPH in general obstetric population and in women at high risk of PPH (e.g. anaemic women)?	92%
14	Cross-cutting	What is the effectiveness of checklists in improving PPH quality of care and PPH-related outcomes compared to current standard of care?	92%
15	Cross-cutting	What is the impact of simulated training with obstetric drills on the quality and outcomes of PPH care, and what are best modalities of simulation?	92%
16	Cross-cutting	What is the effectiveness of a strategy designed for detection and treatment of refractory PPH on morbidity compared to usual care?	92%
17	Implementation	What are the most effective strategies to improve the uptake and sustainment of evidence-based practices for PPH management by women's groups, civil society organizations, and community health care providers?	92%
18	Implementation	What are the most effective and safe strategies for introducing tamponade devices into health service and what are the health, financial, and health system impacts?	92%
19	Implementation	What is the optimal package of labour and childbirth care for reducing PPH-related adverse outcomes?	92%
20	Implementation	What is the optimal package of postnatal care for women who experience PPH, and what is its impact on longer-term maternal and newborn outcomes?	91%
21	Implementation	What is the role of a midwifery-led continuity-of-care model on PPH management and outcomes?	91%
22	Implementation	What is the cost-effectiveness of drug options for PPH prevention and treatment across different settings (low- and high-resource settings)?	90%
23	Innovation	What is the effectiveness, safety, feasibility, and acceptability of alternative administration routes of oxytocin (e.g. inhaled oxytocin) for PPH prevention and treatment?	90%
24	Implementation	What are the most effective implementation strategies to scale up the use of Non-pneumatic Anti-Shock Garment (NASG) for PPH management at the country level?	89%
25	Implementation	What is the impact of introducing antenatal misoprostol distribution programmes on maternal and perinatal outcomes and utilization of health services (particularly facility-based childbirth)?	89%
26	Implementation	What are the most effective strategies for advance antenatal misoprostol distribution for reducing PPH burden and improving outcomes?	89%
27	Cross-cutting	What is the effectiveness of Maternal and Perinatal Death Surveillance and Response programmes in the reduction of maternal deaths due to PPH?	88%
28	Cross-cutting	What is the effectiveness, safety, feasibility, and cost of strategies to improve access of women with PPH to blood and blood replacement products (e.g. fibrinogen concentrate, prothrombin complex concentrate), including in settings without transport capabilities?	88%

29	Implementation	What are the essential health service preconditions for effective and safe introduction of uterine balloon tamponade devices for the treatment of atonic refractory PPH?	88%
30	Innovation	What is the effectiveness and safety of uterine tamponade device as a pre-referral treatment of refractory PPH in basic emergency obstetric and newborn care (BEmONC) setting?	86%
31	Innovation	Can clinical criteria for haemodynamic instability facilitate earlier PPH diagnosis and improved PPH outcomes compared to blood loss measurement alone?	86%
32	Implementation	What is the impact of introducing tamponade devices for refractory PPH treatment on health service organization and resource requirements?	86%
33	Implementation	What is the effectiveness and safety of intrauterine device for PPH treatment when delivered by health professionals other than obstetric specialists (e.g. midwives, general practice doctors) in the context of task-shifting?	85%
34	Cross-cutting	Does antenatal micro- and macronutritient supplementation reduce the risk of PPH in undernourished women?	85%
35	Innovation	What is the comparative effectiveness and safety of tamponade devices in women with refractory PPH during caesarean section in adequately resourced comprehensive emergency obstetric and newborn care (CEmONC) setting?	85%
36	Innovation	What strategies are most effective for engaging the private sector in the development of new PPH medicines, devices, and diagnostics in low- and middle-income countries?	84%
37	Cross-cutting	What is the role of uterotonic agents in the management of secondary PPH (i.e. any significant vaginal bleeding between 24 hours after placental delivery and during the following 6 weeks)?	84%
38	Implementation	What is the effectiveness and feasibility of using a dedicated rapid response team (e.g. "obstetric flying squads") to transport women with PPH from the community to an appropriate health care facility?	83%
39	Innovation	What is the comparative effectiveness of uterine balloon tamponade devices compared to other tamponade interventions (such as suction devices) in the reduction of PPH-related maternal morbidity and mortality?	83%
40	Implementation	What are the skills, competence, and perception regarding PPH diagnosis and treatment among health care professionals other than obstetric specialists (e.g. midwives, general practice doctors, other medical professionals)?	83%
41	Cross-cutting	What is the optimal effective and safe regimen of intravenous oxytocin for PPH prevention?	83%
42	Innovation	What is the effectiveness, safety, and cost of suction tamponade devices (e.g. modified Jada device, Levin gastric tube) compared to standard of care in the treatment of refractory PPH in low- and middle-income countries?	83%

43	Cross-cutting	Can modifications in the current definitions of PPH (e.g. volumetric cut-offs) improve quality and outcomes of PPH care for vaginal and caesarean births?	82%
44	Innovation	What is the impact of universal haemoglobin assay and intravenous iron treatment of anaemic women on PPH-related outcomes?	82%
45	Innovation	What is the optimal dosing regimen, route of administration, effectiveness, and safety of combination of uterotonics, compared to single uterotonic, for the prevention and treatment of PPH?	82%
46	Innovation	What is the impact of intravenous compared to oral iron treatment of anaemia following severe PPH?	81%
47	Cross-cutting	What is the blood loss threshold to trigger first response treatment for PPH in women undergoing vaginal birth and caesarean section?	81%
48	Innovation	What is the comparative effectiveness and safety of purpose-designed compared to improvised uterine balloon tamponade devices in women with refractory PPH during vaginal birth in adequately resourced comprehensive emergency obstetric and newborn care (CEmONC) setting?	81%
49	Cross-cutting	What is the optimal timing for administration of oxytocin for PPH prevention (in relation to cord clamping and placenta delivery)?	81%
50	Implementation	What are the market barriers to investment in new PPH technologies?	81%
51	Cross-cutting	What is the effectiveness and safety of routine use of ergometrine (alone or in combination with oxytocin) compared to oxytocin alone for prevention of PPH during caesarean delivery?	79%
52	Innovation	What is the effectiveness, safety, and cost of sponge haemostatic materials (e.g. XSTAT, Celox gauze) compared to standard of care in the treatment of refractory PPH in low- and middle-income countries?	78%
53	Implementation	What is the role of increased diversity in midwifery workforce (e.g. including under-represented groups) in improving PPH management and outcomes?	78%
54	Cross-cutting	What is the optimal strategy for screening and management of morbidly adherent placenta for prevention of PPH?	78%
55	Cross-cutting	What is the effectiveness and safety of immediate versus delayed manual removal of a retained placenta?	77%
56	Cross-cutting	What is the effectiveness and safety of injectable prostaglandins in the treatment of PPH?	77%
57	Innovation	What is the effectiveness and safety of novel artificial whole blood substitutes in the management of PPH in low- and middle-income countries?	77%
58	Cross-cutting	What is the optimal policy for delaying cord clamping for prevention of PPH and neonatal morbidity?	76%

59	Innovation	What is the impact of introducing intraoperative autotransfusion device (e.g. Hemafuse) on PPH outcomes in settings with limited blood transfusion capacity?	74%
60	Cross-cutting	What are the longer-term effects (on women and breastfed newborns) of tranexamic acid (TXA) when used for PPH treatment?	73%
61	Innovation	What is the effectiveness and safety of early application of aortic compression in women with PPH after vaginal birth compared to usual care?	73%
62	Cross-cutting	What is the effectiveness and safety of routine administration of oxytocin infusion over a period of time (e.g. 2-4 hours) in addition to the standard dose of oxytocin in the prevention of PPH?	73%
63	Innovation	What is the role of bedside tests (e.g. haemoglobin, clotting analysis, thromboelastograph) in the management of PPH?	72%
64	Innovation	What is the comparative effectiveness of uterine compressive suture techniques (e.g. B-Lynch, modified B-Lynch) in surgical conservative treatment of refractory PPH due to uterine atony?	69%
65	Innovation	What is optimal ratio of packed red cells to fresh frozen plasma in the management of massive postpartum haemorrhage?	67%
66	Innovation	What is the effectiveness, safety, and cost of the PPH butterfly device compared to standard of care in the treatment of PPH due to uterine atony?	66%
67	Innovation	What is the comparative effectiveness and safety of interrupted suture uterine closure technique at caesarean section compared to standard (continuous) suturing technique in reducing PPH-related morbidity?	64%
68	Cross-cutting	What is the effectiveness of maternal nipple stimulation during the third stage of labour in the prevention of PPH?	57%
69	Innovation	What is the effectiveness and safety of uterine tamponade in addition to standard care in the prevention of PPH in high risk women?	55%
70	Innovation	Are there genetic mutations associated with PPH (to identify novel targets for drug development)?	51%
71	Innovation	What is the effectiveness of recombinant activated factor VII (rFVIIa) in addition to standard care in the treatment of PPH?	50%
72	Innovation	What is the effectiveness and safety of homeopathic remedies in the prevention of PPH?	27%
