# Supplementary material for: Research agenda for ending preventable maternal deaths from postpartum haemorrhage: a WHO research prioritisation exercise
Source: BMJ Glob Health. 2024 Nov 7;9(11):e015342. doi: 10.1136/bmjgh-2024-015342 (PMC11552529; doi:10.1136/bmjgh-2024-015342)
Supplement: online supplemental file 2 [file bmjgh-9-11-s002.pdf]

Research questions | Top 10 for Innovation

Subcategory Ranking	Section	Suggested research question	Overall Score
1	Innovation	What is the effectiveness and safety of heat-stable carbetocin for PPH treatment in women who received heat-stable carbetocin for PPH prevention?	95%
2	Innovation	What is the comparative effectiveness and safety of alternative routes of tranexamic acid (TXA) in the treatment of PPH?	93%
3	Innovation	What is the effectiveness, safety, feasibility, and acceptability of alternative administration routes of oxytocin (e.g. inhaled oxytocin) for PPH prevention and treatment?	90%
4	Innovation	What is the effectiveness and safety of uterine tamponade device as a pre-referral treatment of refractory PPH in basic emergency obstetric and newborn care (BEmONC) setting?	86%
5	Innovation	Can clinical criteria for haemodynamic instability facilitate earlier PPH diagnosis and improved PPH outcomes compared to blood loss measurement alone?	86%
6	Innovation	What is the comparative effectiveness and safety of tamponade devices in women with refractory PPH during caesarean section in adequately resourced comprehensive emergency obstetric and newborn care (CEmONC) setting?	85%
7	Innovation	What strategies are most effective for engaging the private sector in the development of new PPH medicines, devices, and diagnostics in low- and middle-income countries?	84%
8	Innovation	What is the comparative effectiveness of uterine balloon tamponade devices compared to other tamponade interventions (such as suction devices) in the reduction of PPH-related maternal morbidity and mortality?	83%
9	Innovation	What is the effectiveness, safety, and cost of suction tamponade devices (e.g. modified Jada device, Levin gastric tube) compared to standard of care in the treatment of refractory PPH in low- and middle-income countries?	83%
10	Innovation	What is the impact of universal haemoglobin assay and intravenous iron treatment of anaemic women on PPH-related outcomes?	82%

Zoom | Innovation track explanatory brief #1

Research question

What is the effectiveness and safety of heat-stable carbetocin for PPH treatment in women who received heat-stable carbetocin for PPH prevention?

Score from initial survey

- 6th overall,
- 1st Innovation,
- 95% score

Source & other merged questions

- **Summit participants** (Q211: Carbetocin in the role of a therapeutic agent in the management of PPH. Once we are positioning carbetocin as an effective agent and noninferior to oxytocin in prevention of PPH. Its effect last for 60-120 minutes. It means that it will be administered to all delivering women. In case, a woman bleeds, should we repeat carbetocin? If yes, what should be the time interval? Treatment of PPH by carbetocin is only for those women who have not received carbetocin for prophylaxis?)

Background to this question

- WHO recommends oxytocin for PPH prevention and treatment, but oxytocin requires refrigeration and has limited shelf-life
- Heat-stable carbetocin was found to be as effective to oxytocin for PPH prevention
- Heat-stable carbetocin was added to the WHO Essential Medicines List for PPH prevention in 2019
- However, the effectiveness and safety of heat-stable carbetocin for PPH treatment in women who have already received it for PPH prevention is unknown and there is currently no guidance available on the use of heat-stable carbetocin for PPH treatment
- The objective of this research would be to evaluate the effectiveness and safety of heat-stable carbetocin for PPH treatment

Zoom | Innovation track explanatory brief #2

Research question

What is the comparative effectiveness and safety of alternative routes of tranexamic acid (TXA) in the treatment of PPH?

Score from initial survey

- 12th overall,
- 2nd Innovation,
- 93% score

Source & other merged questions

- **WHO recommendations** (Q42: What are the effects of TXA by other routes of administration (for example, oral, intramuscular, topical, buccal) when used for PPH treatment?) - WHO recommendations on TXA for treatment of PPH; Q45: What are the longer-term effects (on women and breastfed newborns) of TXA when used for PPH treatment? - WHO recommendations on TXA for treatment of PPH)

Background to this question

- WHO recommends tranexamic acid (TXA) for PPH treatment as a slow intravenous (IV) injection over 10 minutes
- However, alternative routes of administration may be easier to administer, especially in lower levels of care
- Intramuscular (IM) injection, or oral administration, have been proposed
- In general bleeding trauma patients, IM TXA was found to be well tolerated and rapidly absorbed
- Future research could compare the effectiveness and safety of different routes of TXA administration for PPH treatment

Zoom | Innovation track explanatory brief #3

Research question

What is the effectiveness, safety, feasibility, and acceptability of alternative administration routes of oxytocin (e.g., inhaled oxytocin) for PPH prevention and treatment?

Score from initial survey

- 23rd overall,
- 3rd Innovation,
- 90% score

Source & other merged questions

- **Pipeline analysis** (Q1: Alternative administration routes of oxytocin that can be used for PPH)

Background to this question

- WHO recommends the use of oxytocin for PPH prevention and treatment. The current route of administration is via intravenous (IV) or intramuscular (IM) injection.
- Alternative routes of administration have been proposed (e.g., inhaled oxytocin) to improve access, especially in remote and resource-constrained settings. Yet little is known about these alternatives.
- Future research could aim to evaluate the effectiveness, safety, feasibility, and acceptability of different routes of oxytocin administration for PPH prevention and treatment. Qualitative research could be used to explore the acceptability of alternative administration routes of oxytocin among women and healthcare providers.

Zoom | Innovation track explanatory brief #4

Research question

What is the effectiveness and safety of uterine tamponade device as a pre-referral treatment of refractory PPH in basic emergency obstetric and newborn care (BEMONC) setting?

Score from initial survey

- 30th overall,
- 4th Innovation,
- 86% score

Source & other merged questions

- **WHO recommendations** (Q28: What is the effectiveness and safety of uterine balloon tamponade when using it as a temporizing measure for treatment of atonic refractory PPH in preparation for referral to a higher level of care, in the reduction of PPH-related severe maternal morbidity and mortality? - WHO recommendations on uterine balloon tamponade for the treatment of postpartum hemorrhage)

Background to this question

- Refractory PPH requires immediate and effective treatment and WHO recommends uterine tamponade devices as a second-line treatment for refractory PPH in settings where advanced care is available.
- There is limited evidence on the use of uterine tamponade devices as pre-referral treatment and future research should contribute to the evidence base on the use of uterine tamponade devices as a pre-referral treatment for refractory PPH in BEMONC settings.
- Qualitative research could be used to explore the acceptability and feasibility of uterine tamponade devices as a pre-referral treatment among women and healthcare providers in BEMONC settings.

Zoom | Innovation track explanatory brief #5

Research question

Can clinical criteria for hemodynamic instability facilitate earlier PPH diagnosis and improved PPH outcomes compared to blood loss measurement alone?

Score from initial survey

- 31th overall,
- 5th Innovation,
- 86% score

Source & other merged questions

- **WHO recommendations** (Q20: Which clinical consequences of blood loss are of greatest value for the diagnosis and treatment of PPH? - WHO recommendations for the prevention and treatment of postpartum hemorrhage, 2012), **Summit participants** (Q90: Rather than focusing on a specific EBL to determine and institute PPH algorithms (EBL being unreliably estimated), is there a set of clinical criteria to apply instead that may lead to better outcomes?)

Background to this question

- Early recognition and intervention are essential for improving outcomes from PPH.
- WHO recommends a blood loss threshold of 500ml or more to diagnose PPH and routine uterine tone assessment to diagnose uterine atony.
- The proposed intervention is to include clinical signs of hemodynamic instability to facilitate earlier diagnosis of PPH.
- Future research could aim to determine whether the use of clinical criteria for hemodynamic instability facilitates earlier PPH diagnosis compared to blood loss measurement alone, and thus improves outcomes.

Source: WHO PPH Summit Research

Zoom | Innovation track explanatory brief #6

Research question

What is the comparative effectiveness and safety of tamponade devices in women with refractory PPH during caesarean section in adequately resourced comprehensive emergency obstetric and newborn care (CEMONC) setting?

Score from initial survey

- 35th overall,
- 6th Innovation,
- 85% score

Source & other merged questions

- **WHO recommendations** (Q32: In adequately resourced settings with good-quality PPH care; what is the safety and comparative effectiveness of different tamponade devices for the treatment of refractory PPH at caesarean section in the reduction of PPH-related maternal morbidity and mortality? - WHO recommendation on uterine balloon tamponade for the treatment of postpartum hemorrhage)

Background to this question

- Tamponade devices are used in the management of refractory PPH.
- There is limited evidence comparing the effectiveness and safety of these devices in women undergoing cesarean section.
- WHO recommends uterine tamponade devices to be used as second-line treatment for refractory PPH for women with ***vaginal*** birth in settings where advanced care is available, but there is no consensus on which device is the most effective and safe.
- The proposed research would compare the effectiveness and safety of different tamponade devices for the management of refractory PPH during cesarean section in CEMONC settings. Future research should also assess the feasibility and acceptability of different tamponade devices.

Zoom | Innovation track explanatory brief #7

Research question

What strategies are most effective for engaging the private sector in the development of new PPH medicines, devices, and diagnostics in low- and middle-income countries?

Score from initial survey

- 36th overall,
- 7th Innovation,
- 84% score

Source & other merged questions

- **Summit participants** (Q53: How can private sector engagement in the development of new PPH medicines, devices and diagnostics targeting most vulnerable populations be increased?; Q16: What are the barriers and facilitators to increasing sustainable and long-term funding to support PPH product R&D?)

Background to this question

- The private sector has a crucial role to play in the development of new PPH medicines, devices, and diagnostics
- However, the private sector may face barriers to engagement in low- and middle-income countries, including limited resources, lack of incentives, and regulatory challenges
- WHO recommends that research and development of PPH medicines, devices, and diagnostics be a priority
- WHO also recommends that engaging the private sector can accelerate innovation and improve access to quality healthcare
- The proposed research would identify and evaluate strategies to improve private sector engagement along with research institutions, regulatory agencies, and civil society groups

Source: WHO PPH Summit Research

Zoom | Innovation track explanatory brief #8

Research question

What is the comparative effectiveness of uterine balloon tamponade devices compared to other tamponade interventions (such as suction devices) in the reduction of PPH-related maternal morbidity and mortality?

Score from initial survey

- 39th overall,
- 8th Innovation,
- 83% score

Source & other merged questions

- **WHO recommendations** (Q30: What is the comparative effectiveness of uterine balloon tamponades compared to other tamponade interventions (such as suction devices) in the reduction of PPH-related maternal morbidity and mortality? - WHO recommendation on uterine balloon tamponade for the treatment of postpartum hemorrhage)

Background to this question

- WHO recommends that uterine balloon tamponade be considered as a second-line treatment for refractory PPH after vaginal birth when initial measures, such as uterotonics, have failed and in settings where advanced care is available
- It is unclear whether other uterine tamponade devices (such as suction devices) are a safe and effective alternative
- The proposed research would compare the effectiveness of uterine balloon tamponade to other uterine tamponade devices in reducing PPH-related maternal morbidity and mortality

Zoom | Innovation track explanatory brief #9

Research question

What is the effectiveness, safety, and cost of suction tamponade devices (e.g., modified Jada device, Levin gastric tube) compared to standard of care in the treatment of refractory PPH in low- and middle-income countries?

Score from initial survey

- 42th overall,
- 9th Innovation,
- 83% score

Source & other merged questions

- **Device prioritization exercise** (Q1-8: In adequately resourced CEMONC setting in an LMIC, what is the (effectiveness/efficacy and safety) of using the (modified Jada device/Levin gastric tube) compared to standard of care in the reduction of PPH-related maternal morbidity and mortality among women who experience (refractory PPH due to uterine atony/PPH due to atony who do not respond to the first response bundle)?)

Background to this question

- There is a lack of evidence on the effectiveness, safety, and cost of using suction tamponade devices for refractory PPH in LMICs
- WHO currently has no specific guidance on the use of suction tamponade devices for refractory PPH
- The current practice for managing refractory PPH in LMICs is limited due to the lack of availability and access to uterine balloon tamponade and other interventions
- Suction tamponade devices may offer a low-cost, easily accessible alternative for the management of refractory PPH
- The proposed research would explore the effectiveness, safety, and cost of uterine suction tamponade devices compared to current standard of care

Source: WHO PPH Summit Research

Zoom | Innovation track explanatory brief #10

Research question

What is the impact of universal hemoglobin assay and intravenous iron treatment of anemic women on PPH-related outcomes?

Score from initial survey

- 44th overall,
- 10th Innovation
- 82% score

Source & other merged questions

- **Summit participants** (Q2:What is the role of universal hemoglobin assay and treatment with IV Iron therapy?)

Background to this question

- WHO recommends identifying and treating anemia in pregnant women
- Oral iron supplements are the most common treatment, as they are generally effective and have few side effects
- For severe anemia, oral supplements may not be effective and intravenous (IV) iron therapy may be more beneficial
- IV iron therapy allows for more rapid absorption and a quicker increase in hemoglobin levels
- Future research could compare the standard of care for PPH prevention with universal hemoglobin assay and intravenous iron treatment of anemic women for improving PPH-related morbidity and mortality, especially in low-income settings

Source: WHO PPH Summit Research

Research questions | Top 10 for Implementation

Subcategory Ranking	Section	Suggested research question	Overall Score
1	Implementation	What are the implementation barriers and facilitators affecting the adoption and use of evidence-based recommendations for PPH management?	99%
2	Implementation	What are the most effective advocacy strategies to improve the uptake and ensure sustainment of evidence-based practices for PPH management at the country level?	96%
3	Implementation	What are the most effective implementation strategies to improve uptake and sustainment of recommended evidence-based interventions for PPH management, including in humanitarian settings?	96%
4	Implementation	What are the optimal strategies to introduce and scale up the use of newer PPH medicines (e.g. heat-stable carbetocin, tranexamic acid) at various levels of care and settings?	96%
5	Implementation	What are the optimal strategies to ensure access to quality-assured PPH medicines (including Universal Health Coverage/Essential Packages for Health Services and Health Benefit Package) in low- and middle-income countries?	95%
6	Implementation	What are the critical components/models for successful introduction and implementation of new/newly recommended medications for PPH management in low- and middle-income countries?	95%
7	Implementation	What is the impact of training programmes for community health workers and paramedics on temporising measures (e.g. uterine massage, external aortic compression, uterine tamponade, Non-pneumatic Anti-Shock Garment) on PPH outcomes?	95%
8	Implementation	What is the effectiveness and cost of pre-service and in-service training programmes for frontline healthcare workers (paramedics, general practice doctors, community health workers, midwives, nurses) to manage and refer women with PPH?	94%
9	Implementation	What are the most effective strategies to improve the uptake and sustainment of evidence-based practices for PPH management by women's groups, civil society organizations, and community health care providers?	92%
10	Implementation	What are the most effective and safe strategies for introducing tamponade devices into health service and what are the health, financial, and health system impacts?	92%

Zoom | Implementation track explanatory brief #1

Research question

What are the implementation barriers and facilitators affecting the adoption and use of evidence-based recommendations for PPH management?

Score from initial survey

- #1 overall
- #1 implementation
- 99% score

Source & other merged questions

- **Summit participants** (Q11: Determine/Assess implementation barriers and enablers of utilization of the WHO recommended PPH prevention and management drugs)

Background to the question

- WHO has issued guidelines for the prevention and management of postpartum hemorrhage, including recommendations for the use of uterotonics, tranexamic acid, and mechanical and surgical interventions
- However, adoption has lagged, hampering use of lifesaving interventions in clinical practice
- There is currently limited research on the best strategies to enable country-level adoption and subsequent use of evidence-based recommendations for PPH management, especially in low- and middle-income countries
- This research will build on existing studies to provide a more comprehensive understanding of the implementation barriers and facilitators affecting the adoption and use of evidence-based recommendations for PPH management

Source: WHO PPH Summit Research

Zoom | Implementation track explanatory brief #2

Research question

What are the most effective advocacy strategies to improve the uptake and ensure sustainment of evidence-based practices for PPH management at the country level?

Score from initial survey

- #2 overall
- #2 implementation
- 96% score

Source & other merged questions

- **Summit participants** (Q113: What are the steps to developing leaders in advocacy to improve Minister of Health responsiveness to PPH needs?)

Background to the question

- Advocacy efforts can influence policy and practice change, but it is not known which advocacy strategies are most effective, and under which circumstances or in which contexts
- There is a need to determine which advocacy strategies are likely to be most influential for promoting the uptake and sustainment of evidence-based practices at the country level
- The proposed research would identify different advocacy strategies and compare their effectiveness for translating evidence-based recommendations into routine clinical practice

Source: WHO PPH Summit Research

Zoom | Implementation track explanatory brief #3

Research question

What are the most effective implementation strategies to improve uptake and sustainment of recommended evidence-based interventions for PPH management, including in humanitarian settings?

Score from initial survey

- #4 overall
- #3 implementation
- 96% score

Source & other merged questions

- **Summit participants** (Q3: What are the best approaches to deliver PPH prevention interventions for women living in humanitarian settings?)

Background to the question

- WHO has guidelines on the prevention and management of PPH, including the use of uterotonics, early recognition and management of PPH, and access to blood transfusion and surgery as needed
- Even with successful adoption at a country level, women often do not receive the recommended interventions, especially in humanitarian settings
- This research would aim to identify the most effective implementation strategies, barriers and facilitators to implementation, assessing the feasibility and acceptability of different implementation strategies, and evaluating the impact of these strategies on the uptake and sustainment of recommended interventions, including in humanitarian settings

Source: WHO PPH Summit Research

Zoom | Implementation track explanatory brief #4

Research question

What are the optimal strategies to introduce and scale up the use of newer PPH medicines (e.g., heat-stable carbetocin, tranexamic acid) at various levels of care and settings?

Score from initial survey

- #5 overall
- #4 implementation
- 96% score

Source & other merged questions

- **Summit participants** (Q27: What are the optimal strategies to implement and scale up the use of 1) carbetocin and 2) tranexamic acid in various levels of care and settings? Particular attention to primary vs secondary vs tertiary care, obstetrician-led vs midwifery-led care settings)

Background to the question

- WHO recommends the use of heat-stable carbetocin and tranexamic acid for the prevention and treatment of PPH
- However, there is limited research and guidance on how to introduce and scale up the use of these medicines in different healthcare settings, including primary health centers, district hospitals, and tertiary care centers
- This research would aim to identify the optimal strategies for introducing and scaling up the use of heat-stable carbetocin and tranexamic acid for the prevention and treatment of PPH; explore the barriers and facilitators to the adoption of these medicines in different settings and levels of care; and provide guidance for policymakers and healthcare providers on the implementation of these strategies

Source: WHO PPH Summit Research

Zoom | Implementation track explanatory brief #5

Research question

What are the optimal strategies to ensure access to quality-assured PPH medicines (including Universal Health Coverage/Essential Packages for Health Services and Health Benefit Package) in low- and middle-income countries?

Score from initial survey

- #7 overall
- #5 innovation
- 95% score

Source & other merged questions

- **Summit participants** (Q155: What are the health economic consequences of poor quality PPH medicines (and are there outstanding research questions that need to be answered to inform credible HE models to answer this question)?; Q124: What are the most effective strategies to assist countries to procure and effectively supply appropriate quality-assured PPH medicines?; Q151: Is there a (multiplier) role for UHC (including Essential Packages (for Health Services and Health Benefit Package) in accelerating access to PPH medicines and interventions?)

Background to the question

- WHO emphasizes the importance of Universal Health Coverage (UHC)/Essential Packages for Health Services (EPHS) and Health Benefit Package (HBP) approaches in ensuring access to essential medicines, including quality-assured PPH medicines
- Ensuring access to quality-assured PPH medicines is often difficult in LMICs due to inadequate supply chain management and lack of investment
- This research would aim to identify the barriers to access quality-assured PPH medicines in LMICs; the optimal strategies to ensure access to quality-assured PPH medicines in LMICs; and provide guidance to policymakers and healthcare providers on the implementation of these strategies

Source: WHO PPH Summit Research

Zoom | Implementation track explanatory brief #6

Research question

What are the critical components/models for successful introduction and implementation of new/newly recommended medications for PPH management in low- and middle-income countries?

Score from initial survey

- #8 overall
- #6 implementation
- 95% score

Source & other merged questions

- **Summit participants** (Q11; What are the critical components/model for successful introduction and implementation of the new/newly recommended PPH prevention and management drugs in LMICs?)

Background to the question

- The introduction and implementation of new and newly recommended medications for postpartum hemorrhage (PPH) management in low- and middle-income countries (LMICs) is often challenging due to various barriers, including limited resources, lack of trained healthcare providers, and inadequate supply chain management
- WHO emphasizes the importance of collaboration between different stakeholders, including policymakers, healthcare managers and providers, to ensure the successful implementation of new medications
- This research would aim to identify the critical components and models for successful introduction and implementation of new PPH medications in LMICs and provide guidance to policymakers and healthcare providers on the implementation of these components and models

Source: WHO PPH Summit Research

Zoom | Implementation track explanatory brief #7

Research question

What is the impact of training programmes for community health workers and paramedics on temporising measures (e.g., uterine massage, external aortic compression, uterine tamponade, Non-pneumatic Anti-Shock Garment) on PPH outcomes?

Score from initial survey

- #9 overall
- #7 implementation
- 95% score

Source & other merged questions

- **Summit participants** (Q128: Will systematic training in uterine massage and external aortic compression among ambulance personnel and other community healthcare workers lead to decreased morbidity and mortality among women giving birth outside of hospitals?), FIGO 2012 (Q2: Further assess options for treatment of PPH in lower-level (basic EmOC) facilities—in particular, uterine tamponade and the antishock garment)

Background to the question

- Timely management of PPH is critical to reducing the associated morbidity and mortality and community health workers (CHWs) and paramedics can play an important role, especially in resource-limited settings
- WHO provides guidelines for the prevention and management of PPH, including the use of temporizing measures
- The current training programs on CHWs and paramedics in the use of temporizing measures for PPH management is unclear
- This research would aim to assess the impact of training programs on the use of temporizing measures for PPH management by CHWs and paramedics and evaluate the effect of the use of temporizing measures on PPH outcomes in the community

Zoom | Implementation track explanatory brief #8

Research question

What is the effectiveness and cost of pre-service and in-service training programmes for frontline healthcare workers (paramedics, general practice doctors, community health workers, midwives, nurses) to manage and refer women with PPH?

Score from initial survey

- #10 overall
- #8 implementation
- 94% score

Source & other merged questions

- **Summit participants** (Q166: Role of training programs directed towards midwives and health care workers in effective handling of PPH)

Background to the question

- Frontline healthcare workers (paramedics, general practice doctors, community health workers, midwives, nurses) play a crucial role in the timely and effective management of PPH
- The current practice of PPH management varies widely across different settings, and the effectiveness of training programs for frontline healthcare workers to manage and refer women with PPH is not well established
- The proposed intervention is the implementation of pre-service and in-service training programs for frontline healthcare workers to manage and refer women with PPH
- The study will evaluate the effectiveness, cost-effectiveness, and key factors that influence the success of these programs of these programs

Zoom | Implementation track explanatory brief #9

Research question

What are the most effective strategies to improve the uptake and sustainment of evidence-based practices for PPH management by women's groups, civil society organizations, and community health care providers?

Score from initial survey

- #17 overall
- #9 implementation
- 92% score

Source & other merged questions

- **Summit participants** (Q9: Optimal mechanism for engaging women's groups, community health workers and civil society in strategies to reduce deaths from PPH)

Background to the question

- Women's groups, civil society organizations, and community health care providers have the potential to play a crucial role in improving the uptake and sustainment of evidence-based practices
- Proposed interventions may include the development and implementation of targeted training and capacity-building programs, the use of community-based approaches to promote awareness and demand for evidence-based practices, and the development of supportive policy frameworks
- The aim of this research would be to identify the most effective strategies to improve the uptake and sustainment of evidence-based practices for PPH management by women's groups, civil society organizations, and community health care providers in low- and middle-income countries

Source: WHO PPH Summit Research

Zoom | Implementation track explanatory brief #10

Research question

What are the most effective and safe strategies for introducing tamponade devices into health service and what are the health, financial, and health system impacts?

Score from initial survey

- #18 overall
- #10 implementation
- 92% score

Source & other merged questions

- **WHO recommendations** (Q33: In adequately resourced settings with good-quality PPH care: What is the most effective modality for training and assuring competency in the use of uterine balloon tamponade? - WHO recommendation on uterine balloon tamponade for the treatment of postpartum haemorrhage)

Background to the question

- WHO recommends the use of tamponade devices for the management of refractory PPH after vaginal birth, but only a small number of health systems have incorporated these devices into their guidelines
- The use of tamponade devices for managing PPH is increasing, and it is essential to understand the most effective and safe strategies for introducing these devices into health services
- Future research could identify the best strategies for introducing tamponade devices into health services, taking into account factors such as provider training, cost, and availability

Source: WHO PPH Summit Research

Research questions | Top 10 for Cross-Cutting

Subcategory Ranking	Section	Suggested research question	Overall Score
1	Cross-cutting	What is the effectiveness of a strategy of early detection and first response treatment using a bundle of recommended interventions for improving PPH-related outcomes?	96%
2	Cross-cutting	What is the effectiveness and safety of a diagnostic algorithm (e.g. shock index) and early detection strategies (e.g. Modified Early Obstetric Warning Score) in improving clinical detection and management of PPH?	93%
3	Cross-cutting	What is the effectiveness and safety of tranexamic acid (TXA) in the prevention of PPH in general obstetric population and in women at high risk of PPH (e.g. anaemic women)?	92%
4	Cross-cutting	What is the effectiveness of checklists in improving PPH quality of care and PPH-related outcomes compared to current standard of care?	92%
5	Cross-cutting	What is the impact of simulated training with obstetric drills on the quality and outcomes of PPH care, and what are best modalities of simulation?	92%
6	Cross-cutting	What is the effectiveness of a strategy designed for detection and treatment of refractory PPH on morbidity compared to usual care?	92%
7	Cross-cutting	What is the effectiveness of Maternal and Perinatal Death Surveillance and Response programmes in the reduction of maternal deaths due to PPH?	88%
8	Cross-cutting	What is the effectiveness, safety, feasibility, and cost of strategies to improve access of women with PPH to blood and blood replacement products (e.g. fibrinogen concentrate, prothrombin complex concentrate), including in settings without transport capabilities?	88%
9	Cross-cutting	Does antenatal micro- and macronutritient supplementation reduce the risk of PPH in undernourished women?	85%
10	Cross-cutting	What is the role of uterotonic agents in the management of secondary PPH (i.e. any significant vaginal bleeding between 24 hours after placental delivery and during the following 6 weeks)?	84%

Zoom | Cross-cutting track explanatory brief #1

Research question

What is the effectiveness of a strategy of early detection and first response treatment using a bundle of recommended interventions for improving PPH-related outcomes?

Score from initial survey

- 3rd overall score
- 1st cross-cutting,
- 96% score

Source & other merged questions

- **Summit participants** (Q35: What is the effectiveness of the initial bundle for PPH treatment compared to non-standardized treatment in Vaginal and C-section deliveries?)

Background to this question

- Early detection and initiation of first-response treatment can dramatically improve PPH-related outcomes
- A WHO CHNRI prioritization exercise called for research into innovative approaches to improve monitoring of women postpartum for the early diagnosis and treatment of PPH – the question was ranked #2 discovery question for the whole of maternal health
- Bundling interventions has been proposed as an implementation approach to improve early detection and expedite first-response treatment
- The proposed research would assess the effectiveness of this implementation strategy compared to standard of care
- Additional evidence is needed to assess the feasibility, acceptability, and cost of scaling the bundle approach

Zoom | Cross-cutting track explanatory brief #2

Research question

What is the effectiveness and safety of a diagnostic algorithm (e.g. shock index) and early detection strategies (e.g. Modified Early Obstetric Warning Score) in improving clinical detection and management of PPH?

Score from initial survey

- 11th overall,
- 2nd cross-cutting,
- 93% score

Source & other merged questions

- **WHO CHNRI 2015** (Q13: Evaluate the effectiveness and safety of a diagnostic algorithm to improve the clinical detection and management of PPH, on maternal health outcomes; Q5: Develop and evaluate interventions to improve the quality of postnatal care on the detection and management of postpartum haemorrhage to improve maternal health outcomes)

Background to this question

- Early detection and prompt management of PPH are essential components of quality maternal health care to prevent adverse outcomes
- There is limited evidence on the use of diagnostic algorithms and early detection strategies for PPH
- The proposed research would assess the use of diagnostic algorithms including tools such as the Obstetric Shock Index, and strategies, such as the Modified Early Obstetric Warning Score (MEOWS), to improve early detection of PPH

Source: WHO PPH Summit Research

Zoom | Cross-cutting track explanatory brief #3

Research question

What is the effectiveness and safety of tranexamic acid (TXA) in the prevention of PPH in general obstetric population and in women at high risk of PPH (e.g. anemic women)?

Score from initial survey

- 13th overall,
- 3rd cross-cutting,
- 92% score

Source & merged questions

- **Summit participants** (Q42: Based on available knowledge should be promote hemoglobin estimation on admission and advocate prophylactic, oxytocin and tranexamic acid for those with moderate and severe anemia)

Background to this question

- Tranexamic acid (TXA) has been shown to reduce bleeding in a variety of surgical and traumatic settings and is now recommended by WHO for PPH treatment and included in the Essential Medicines List
- In non-obstetric settings, TXA has been shown to help prevent excessive bleeding
- It's safety and effectiveness for PPH prevention is unknown
- The proposed research would assess the use of TXA before or immediately after delivery to prevent PPH, particularly in high-risk populations (e.g. anemic women)

Zoom | Cross-cutting track explanatory brief #4

Research question

What is the effectiveness of checklists in improving PPH quality of care and PPH-related outcomes compared to current standard of care?

Score from initial survey

- 14th overall,
- 4th cross-cutting,
- 92% score

Source & merged questions

- **Summit participants** (Q184: utility of checklists of HEM readiness for each site (can vary depending in level of care))

Background to this question

- Checklists are a potential intervention that can improve the quality of care for PPH, but the evidence is lacking on effectiveness of checklists in improving PPH quality of care and PPH-related outcomes
- To bridge the gap between evidence and practice, WHO created the Safe Childbirth Checklist, a practical tool to assist birth attendants in planning for and performing a more complete bundle of 28 essential birth practices including recommended practices related to PPH
- Several professional societies have published guidelines that recommend the use of checklists for PPH management
- The proposed intervention is the use of a checklist to guide PPH management, which may include items such as timely administration of uterotonics, quantification of blood loss, and escalation of care as needed

Zoom | Cross-cutting track explanatory brief #5

Research question

What is the impact of simulated training with obstetric drills on the quality and outcomes of PPH care, and what are best modalities of simulation?

Score from initial survey

- 15th overall,
- 5th cross-cutting,
- 92% score

Source & merged questions

- **Summit participants** (Q33: Will simulator training improve outcome in PPH-related maternal mortality and birth asphyxia?)

Background to this question

- Simulation-based training is one strategy for improving the clinical skills and teamwork of healthcare professionals
- WHO recommends the use of simulation-based training to improve quality and patient safety
- The proposed research would assess the utility of simulation-based training on PPH preparedness and response
- The simulation training should reflect real-world clinical scenarios and include both technical and non-technical skills training (e.g., teamwork, communication, leadership)
- Specifically, can assess effectiveness of different simulation modalities; explore factors that influence skill acquisition and transfer; investigate impact on clinical outcomes and patient experience; identify best practices for the design, delivery, and evaluation of simulation-based training for PPH care

Zoom | Cross-cutting track explanatory brief #6

Research question

What is the effectiveness of a strategy designed for detection and treatment of refractory PPH on morbidity compared to usual care?

Score from initial survey

- 16th overall,
- 6th cross-cutting,
- 92% score

Source & merged questions

Summit participants (Q160; Is a complex intervention of treatment approaches for refractory PPH effective to reduce severe morbidity in women with PPH?; Q129: Is a complex intervention of treatment approaches for refractory PPH feasible to be implemented in secondary level hospitals in LMICs?; Q38: What is the most effective treatment bundle for refractory PPH?; Q140: What innovations are most useful to address refractory PPH? e.g., tamponade devices, clotting agents)

Background to this question

- Refractory PPH is particularly challenging to manage and can lead to severe morbidity, including the need for transfusion, hysterectomy, and other surgical interventions
- While several interventions exist to manage refractory PPH, there is a lack of high-quality evidence comparing different strategies for their effectiveness
- The main objective of future research should be to develop and assess the effectiveness of a standardized approach for detection and treatment of refractory PPH (e.g., bundle, algorithm)

Source: WHO PPH Summit Research

Zoom | Cross-cutting track explanatory brief #7

Research question

What is the effectiveness of Maternal and Perinatal Death Surveillance and Response programmes in the reduction of maternal deaths due to PPH?

Score from initial survey

- 27th overall,
- 7th cross-cutting,
- 88% score

Source & merged questions

- **Summit participants** (Q37: Effectiveness of Maternal and Perinatal Death Surveillance and Response in reduction of maternal deaths due to PPH)

Background to this question

- Maternal and Perinatal Death Surveillance and Response (MPDSR) programs have been implemented in some countries to track maternal and perinatal deaths, identify cause of death, and develop and implement strategies to prevent future deaths
- However, there is a need to determine the impact of these programs to encourage universal adoption
- WHO recommends the establishment of MPDSR programs and published guidelines on how to establish and strengthen them
- The objectives of this research would be to identify the key components of MPDSR programs that contribute to their effectiveness; determine the impact of MPDSR programs on maternal mortality due to PPH; and identify the factors that facilitate or hinder the implementation of MPDSR programs in different settings, including the availability of resources, the involvement of key stakeholders, and the political and social context

Zoom | Cross-cutting track explanatory brief #8

Research question

What is the effectiveness, safety, feasibility, and cost of strategies to improve access of women with PPH to blood and blood replacement products (e.g., fibrinogen concentrate, prothrombin complex concentrate), including in settings without transport capabilities?

Score from initial survey

- 28th overall,
- 8th cross-cutting,
- 88% score

Source & merged questions

- **Summit participants** (Q102: (road accidents, work accidents) represent the most part of the blood demand: do synergies exist to maximize the use of blood among these two sectors?; Q195: Can a well-planned human blood bank system of volunteers help reduce the deaths due to PPH where blood transfusion can save a mother's life?) **RCOG 2016** (Q4: Studies are required to determine the role of fibrinogen concentrate in the management of PPH; Q5: The role of prothrombin complex concentrate in the management of PPH requires evaluation)

Background to this question

- Safe blood and blood replacement products are critical tools for managing PPH, particularly when severe, but access is challenging in many settings, and there is limited guidance on how to improve access
- Future research should aim to evaluate the effectiveness, safety, feasibility, and cost of strategies to improve access to blood and blood replacement products for women with PPH, including in settings without transport capabilities
- There are ongoing initiatives to improve access to blood and blood products in low-resource settings, including the WHO's Safe Blood for Africa program and the Blood Transfusion Safety project of the African Society for Blood Transfusion

Zoom | Cross-cutting track explanatory brief #9

Research question

Does antenatal micro- and macronutrient supplementation reduce the risk of PPH in undernourished women?

Score from initial survey

- 34th overall,
- 9th cross-cutting,
- 85% score

Source & merged questions

- **Summit participants** (Q30: Is there a relationship between micronutrient status intake and level and the risk of PPH?; Q91: Can PPH be prevented or the risk for PPH be reduced in underweight, nutrient deficient women when supported with a high energy, high protein, vitamin and mineral enriched supplement during the third trimester? (LMIC))

Background to this question

- Anemia is an important underlying contributor to PPH and adverse outcomes from PPH and WHO recommends provision of iron and folic acid supplementation to all pregnant women to prevent maternal anemia
- However, there is currently no specific guidance on the use of other macro- and micronutrient supplementation to prevent PPH in undernourished women
- The proposed research would evaluate the impact of providing additional micro- and macronutrient supplementation to undernourished pregnant women to reduce the risk of PPH

Zoom | Cross-cutting track explanatory brief #10

Research question

What is the role of uterotonic agents in the management of secondary PPH (i.e. any significant vaginal bleeding between 24 hours after placental delivery and during the following 6 weeks)?

Score from initial survey

- 37th overall,
- 10th cross-cutting,
- 84% score

Source & merged questions

- **Summit participants** (Q88: Use of ergometrine and oxytocin to address PPH due to uterine atony), RCOG 2016 (Q6: RCTs are required to investigate the role of uterotonic agents (misoprostol and ergometrine) in the management of secondary PPH)

Background to this question

- WHO recommends uterotonic agents, such as oxytocin, as the first-line treatment for primary PPH (first 24 hours after birth)
- However, there is limited guidance on the use of these agents for the management of secondary PPH (beyond the first 24 hours after birth)
- The current practice for the management of secondary PPH is varied, and there is a lack of consensus on the use of uterotonic agents
- The aim of this research would be to determine the effectiveness and safety of uterotonic agents including oxytocin, misoprostol, and others in the management of secondary PPH

Source: WHO PPH Summit Research
